# 18 F-FDG PET/CT in Extensive Graft-Versus-Host Disease of the Gastrointestinal Tract Following Autologous Stem Cell Transplantation

**DOI:** 10.3390/diagnostics8040072

**Published:** 2018-10-15

**Authors:** Danijela Dejanovic, Annemarie Amtoft, Annika Loft

**Affiliations:** Department of Clinical Physiology, Nuclear Medicine and PET, Rigshospitalet, University of Copenhagen, 1165 Copenhagen, Denmark; Annemarie.gjelstrup.amtoft@regionh.dk (A.A.); Annika.loft.jakobsen@regionh.dk (A.L.)

**Keywords:** FDG, PET/CT, autologous, HSCT, gastro-intestinal tract, GVHD, Hodgkin’s, lymphoma

## Abstract

Graft-versus-host-disease (GVHD) following stem cell transplantation (SCT) is a common complication in patients that have undergone allogenic SCT but rare in recipients of autologous SCT. Gastro-intestinal tract (GIT)-GVHD can be difficult to diagnose due to non-specific symptoms such as fever, nausea, diarrhea, and vomiting; a histological confirmation is therefore required. Here, we present the findings of a whole-body ^18^FDG PET/CT with extensive and multifocal involvement of the GIT in a patient that developed severe acute GVHD 93 days post autologous SCT for Hodgkin’s lymphoma. PET and CT findings included characteristic patterns of bowel inflammation with bowel wall thickening, mural stratification and enhancement with high FDG-uptake of the involved regions, as well as typical extra intestinal findings such as ascites, engorgement of the vasa recti and stranding of the mesenteric fat. Although, the above-mentioned findings are not exclusive to GIT-GVHD and can be seen in other settings of inflammatory bowel disease such as enterocolitis or Mb Crohn our findings were used for targeted biopsy that confirmed acute GIT-GVHD. This case demonstrates that ^18^F-FDG-PET/CT can be a valuable non-invasive tool in mapping the activity and distribution of intestinal GVHD and direct for targeted biopsies of involved regions.

**Figure 1:**
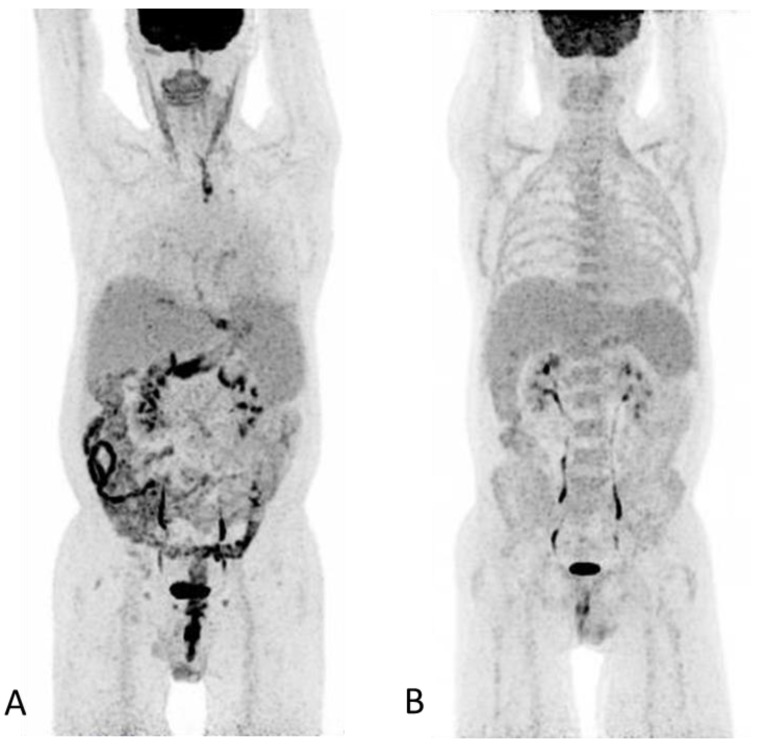
(**A**) An ^18^F-FDG PET/CT, with intravenous and oral contrast enhancement, was performed in a 50-year-old man 93 days post autologous SCT for relapse of Hodgkin’s lymphoma. The PET maximum intensity projection (MIP) image shows discontinuous pathological FDG-uptake throughout the GIT extending from the upper esophagus to the anal canal involving the cardia region, ventricle, and segments of the small-and large bowel including the rectum. The PET findings are consistent with an inflammatory response with normal physiological FDG-uptake in unaffected segments of the GIT. At the time of scanning, the patient had severe diarrhea, fever, nausea and reported weight loss but no skin lesions. The clinical presentation with non-specific symptoms and and that the patient was a recipient of autologous SCT rendered infection as a probable diagnosis. However, repeated blood and stools tests for bacterial, common viral and fungal agents were negative. Diagnosis was based on rectal biopsies that showed epithelial cell apoptosis indicative of GVHD according to previously described criteria [1]. No infectious agents were present. Despite intensive treatment the patient succumbed due to complications following sepsis. (**B**) Normal ^18^F-FDG PET/CT in the same patient performed immediately post-autologous SCT. Amid the two scans it is evident that the spleen has increased in size resulting in splenomegaly (**A**). Splenomegaly has previously been described as an extra intestinal finding in acute GVHD-GIT [2]. Clinically, GVHD is divided into acute or chronic GVDH with acute GVHD developing within the first 100 days post-SCT and chronic GVHD as occurring beyond the first 3 months [3]. GVHD is a leading cause of morbidity and mortality in recipients of allogenic SCT. Auto-GVHD has the same clinical and histological presentation as allo-GVHD but is often observed as milder, self-limited and less frequent [4,5,6]. The standard treatment of acute GVHD is steroids, however, acute auto-GVHD can often resolve without treatment, although, fatal cases have been reported [4,5,6,7]. The primary affected organs are the skin, liver and GIT [8], if all three organs are involved diagnosis could be made on clinical grounds alone. Isolated GVHD-GIT is much more challenging to diagnose due its often non-specific symptoms such as abdominal cramping, nausea and vomiting, voluminous and often bloody diarrhea and fever that in the setting of post-HSCT could be treatment related or infectious. ^18^F-FDG PET/CT has been proposed as a non-invasive imaging modality in assessing intestinal GVHD, map its localization and monitor treatment response [9,10,11]. Here, we present an ^18^F-FDG PET/CT scan where many of the most common reported CT and PET/CT features of GIT-GVHD were present [2,9]. Common PET/CT features of bowel inflammation are non-specific and could represent inflammatory bowel disease such as enterocolitis, Mb Crohn or ulcerative colitis the discontinuous pattern and extent (from esophagus to rectum) of GIT involvement led to us to suggest GVHD as a differential diagnosis. However, caution must be taken for false positive results with/or without accompanied morphological changes. A previous study reported false-positive results for acute-GVHD related to daily metformin treatment [10]. Other pathological underlying causes for pathological FDG-uptake in the GIT must be considered, such as malignancy, benign neoplasms or inflammation due to esophagitis or gastritis [12]. Furthermore, physiological FDG-uptake within the normal GIT is highly variable and findings should be interpreted with care [12]. In this case, many of the possible differential diagnosis could be excluded due to a recently previous FDG-PET/CT scan (**B**) available for comparison which rendered a new malignancy as improbable and the patient had no medical history of inflammatory bowel disease or metformin treated diabetes.

**Figure 2:**
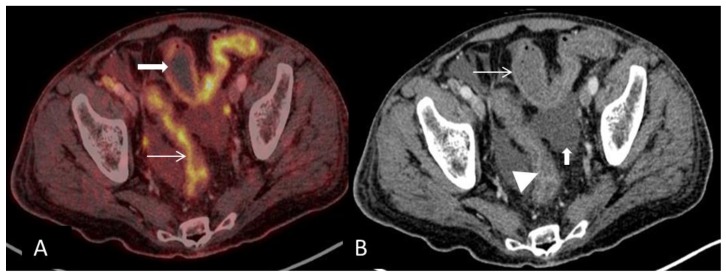
(**A**) Axial PET and CT fused image shows pathological high FDG-uptake in the large-bowel in two different patterns corresponding to the lumen (arrow) and in the outer wall (bold arrow) respectively and is consistent with an inflammatory response. (**B**) CT scan shows mural stratification (i.e., visualization of two or three different layers of the bowel wall) of the thickened large-bowel wall (defined as >3 mm) with mucosal (arrowhead) and serosal enhancement (arrow). The PET/CT findings described here are typical for acute GVHD-GIT or inflammatory bowel disease. Ascites is a common finding in acute-GVHD and has been reported to occur in 45% of affected patients (bold arrow) [2].

**Figure 3:**
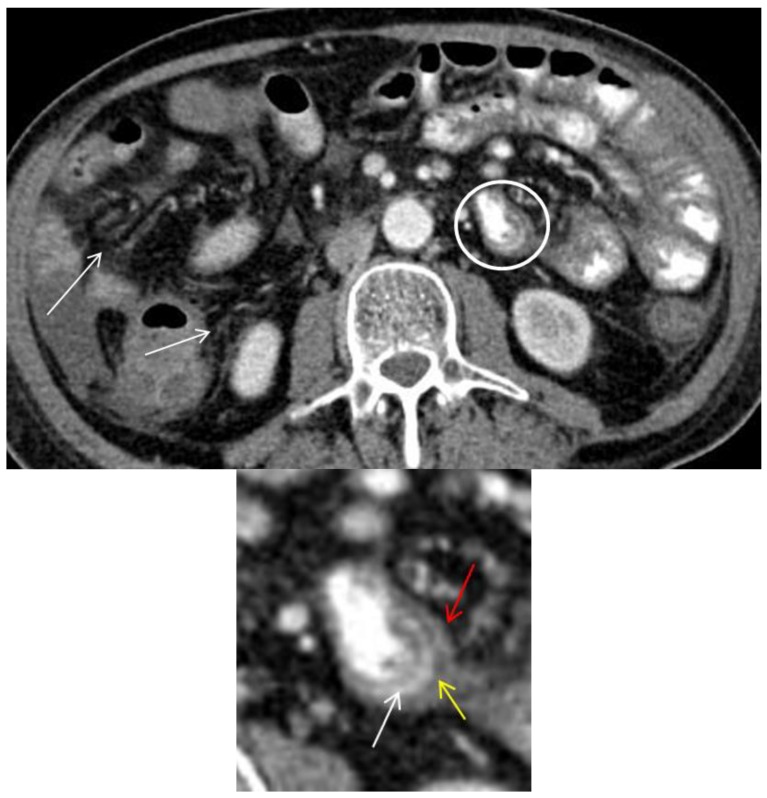
Axial CT image shows circumferential small bowel wall thickening (>3 mm) with trilaminar mural stratification in the small-bowel (circle and magnified image) with serosal enhancement (red arrow), low intramural attenuation (yellow arrow) and mucosal enhancement (white arrow) [2,13]. Low intramural attenuation can represent edema, inflammatory infiltrate or fat [13]. Stranding of the mesenteric fat is often observed in GIT-GVHD [2,6] (arrows). Mural stratification is a non-specific sign of bowel inflammation and has been shown to correlate with clinically active disease as opposed of a more homogenously enhancement due to fibrosis [14,15]. In this patient, three different patterns of mural stratification and mural enhancement were present in separate segments (Figure 2 and Figure 3).

**Figure 4:**
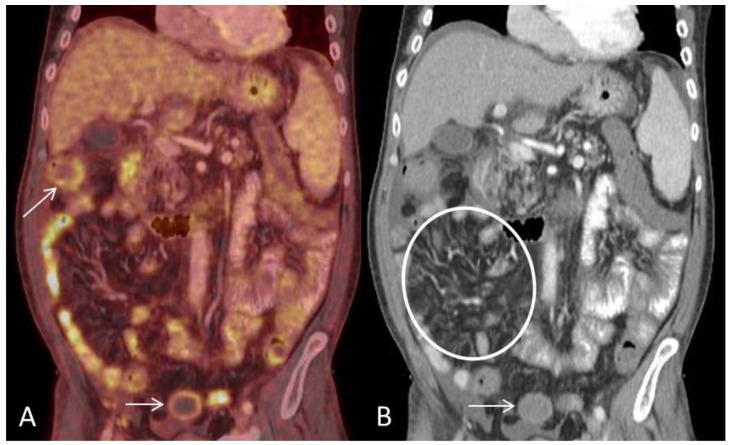
(**A**) Coronal PET and CT fused image demonstrates multifocal segmental pathological FDG-uptake in small-and large bowel loops. Circumferential mural pathological FDG-uptake in the large bowel (arrows). (**B**) Coronal CT shows engorgement of the vasa recta (circle) and mural enhancement of the large bowel (arrow). Ascites is present on the surface of the liver. Engorgement of the vasa recta is due to increased blood-flow in small arteries of inflamed bowel segments and has been reported as the most consistent extraintestinal finding in acute GIT-GVHD [2].

**Figure 5:**
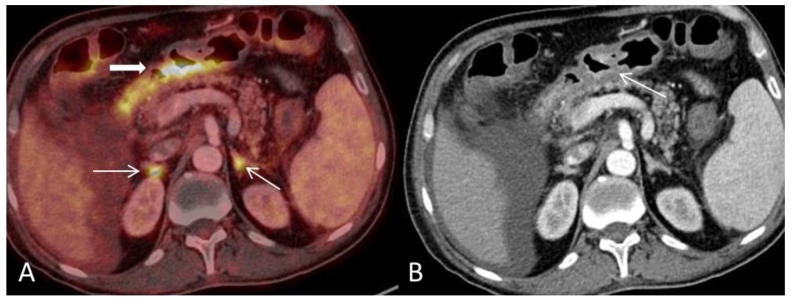
(**A**) Axial PET and CT fused image shows pathological FDG-uptake in the wall of antrum and to a lesser degree in pylorus (bold arrow). Both adrenal glands are seen with high FDG-uptake with no morhological changes, the foci were intepreted as physiological and most likely stress-related (arrows); (**B**) Axial CT shows thickening of the antrum wall with mucosal enhancement (arrow). Ascites surrounding the liver is also present.
